# A comparative study of the mechanical and thermal properties of defective ZrC, TiC and SiC

**DOI:** 10.1038/s41598-017-09562-x

**Published:** 2017-08-24

**Authors:** M. Jiang, J. W. Zheng, H. Y. Xiao, Z. J. Liu, X. T. Zu

**Affiliations:** 1 School of Physical Electronics, University of Electronic Science and Technology of China, Chengdu, 610054 China; 2 Department of Physics, Lanzhou City University, Lanzhou, 730070 China; 3 Institute of Fundamental and Frontier Sciences, University of Electronic Science and Technology of China, Chengdu, 610054 China

## Abstract

ZrC and TiC have been proposed to be alternatives to SiC as fuel-cladding and structural materials in nuclear reactors due to their strong radiation tolerance and high thermal conductivity at high temperatures. To unravel how the presence of defects affects the thermo-physical properties under irradiation, first-principles calculations based on density function theory were carried out to investigate the mechanical and thermal properties of defective ZrC, TiC and SiC. As compared with the defective SiC, the ZrC and TiC always exhibit larger bulk modulus, smaller changes in the Young’s and shear moduli, as well as better ductility. The total thermal conductivity of ZrC and TiC are much larger than that of SiC, implying that under radiation environment the ZrC and TiC will exhibit superior heat conduction ability than the SiC. One disadvantage for ZrC and TiC is that their Debye temperatures are generally lower than that of SiC. These results suggest that further improving the Debye temperature of ZrC and TiC will be more beneficial for their applications as fuel-cladding and structural materials in nuclear reactors.

## Introduction

Cubic silicon carbide (3C-SiC), titanium carbide (TiC) and zirconium carbide (ZrC) are taken as attractive candidates for a variety of applications, including the structural component in fusion reactors, the fuel cladding material for gas-cooled fission reactors and the pressure vessels in Tristructural-isotropic fuel^1–4^, due to their excellent physical and chemical properties such as high thermal conductivity, good mechanical strength, outstanding corrosion as well as fission product retention ability^5–9^. In these applications, carbide materials are exposed to different kinds of radiation environment, i.e., electron^10^, neutron^11^ and ion^12^ irradiation. Consequently, atomic defects, lattice disorder, and even structural amorphization are induced, and their mechanical properties and performances may be affected significantly. For example, radiation damage accumulation leads to a significant decrease in the bulk and Young’s moduli of 3C-SiC by 23% and 29%, respectively^13^. Therefore, it is of crucial importance to investigate the phase stability and the effect of point defects on the thermo-physical properties of carbides.

In the past decades, the radiation damage effect of 3C-SiC has been widely studied. Inui *et al*. investigated the electron irradiation-induced crystalline-to-amorphous (C-A) transition of 3C-SiC as a function of irradiation temperature employing the ultra-high-voltage electron microscopy, and they found that C-A transition can be induced at temperatures below 340 K^10^. The dose of electrons required for the C-A transition is essentially constant at temperatures below 250 K^10^. Snead *et al*. reported that under neutron irradiation 3C-SiC could be amorphized, and the swelling of 3C-SiC increased with the increasing neutron dose until it was saturated at the temperature region between 423 and 1073 K^11^. Weber *et al*. investigated the C-A transition of 3C-SiC using high-voltage electron microscopy with 1.5 MeV Xe^+^ ions over the temperature range from 40 to 550 K, and they found that the samples completely amorphized at 0.34 displacement per atom (dpa) at 0 K^12^. Recently, ZrC and TiC have been proposed to be promising alternatives to SiC due to their higher thermal conductivities at high temperatures and stronger tolerance to irradiation-induced-amorphization. Pellegrino *et al*. carried out experimental studies of the responses of SiC, TiC and ZrC to 1.2 MeV Au^+^ irradiation at room temperature, and found that SiC was amorphized at low Au^+^ fluences, whereas TiC and ZrC maintained their crystalline structure at higher Au^+^ fluences^9^. In our previous studies, an *ab initio* molecular dynamics simulation of low energy radiation responses of ZrC, TiC and SiC has been performed, and it was shown that carbon displacements are dominant in the cascade events and carbon disorder occurs more easily in SiC than ZrC and TiC, which may be responsible for the C-A transition of SiC under irradiation. The stronger radiation tolerance of ZrC and TiC than SiC can be ascribed to their different electronic structures, i.e., the <Ti-C> and <Zr-C> bonds are a mixture of covalent, metallic, and ionic character, whereas the <Si-C> bond is mainly covalent^8^.

In the meantime, researchers have paid attention to the effect of radiation damage on the mechanical properties of carbide materials. Gao *et al*. have employed the molecular dynamics (MD) simulation to investigate the accumulation of radiation damage of 3C-SiC due to the 10 keV Si displacement cascades and analyzed the changes in elastic constants and bulk modulus as the function of dose^13^. They proposed that point defects and small clusters may contribute more significantly to the changes in mechanical properties than topological disorder^13^. In their studies, the classical Tersoff-type potentials were employed and the electronic effects were not modeled. Xi *et al*. investigated the effect of various point defects on the volume swelling and elastic modulus of SiC employing the MD method, and reported that the Young’s modulus of SiC increased with the existence of C_Si_ (carbon occupying the silicon lattice site) antisite defect, while other kinds of point defects had negative influences on the change of Young’s modulus^14^. Li *et al*. carried out MD simulation of the thermal properties of SiC containing carbon and silicon vacancy defects, and found that the thermal conductivity decreased pronouncedly at high concentration of carbon or silicon vacancy defect, i.e., 0.5%^15^. As for the transition metal carbides, the investigations of how point defects affect the mechanical and thermal properties are very few. Dridi *et al*. employed the first-principle calculations to study the effect of vacancies on the structural properties in substoichimetric TiC_x_ (x = 0.25, 0.5 and 0.75) and found that the bulk modulus decreased with the increasing concentration of carbon vacancy^16^. Wang *et al*. compared the mechanical properties of TiC and TiC_0.25_N_0.75_ by using the first-principles calculations, and they proposed that the introduction of nitrogen significantly increased the bulk, shear and Young’s moduli of TiC^17^. Obviously, the presence of point defects has non-negligible effects on the thermo-physical properties of carbides.

**Figure 1:**
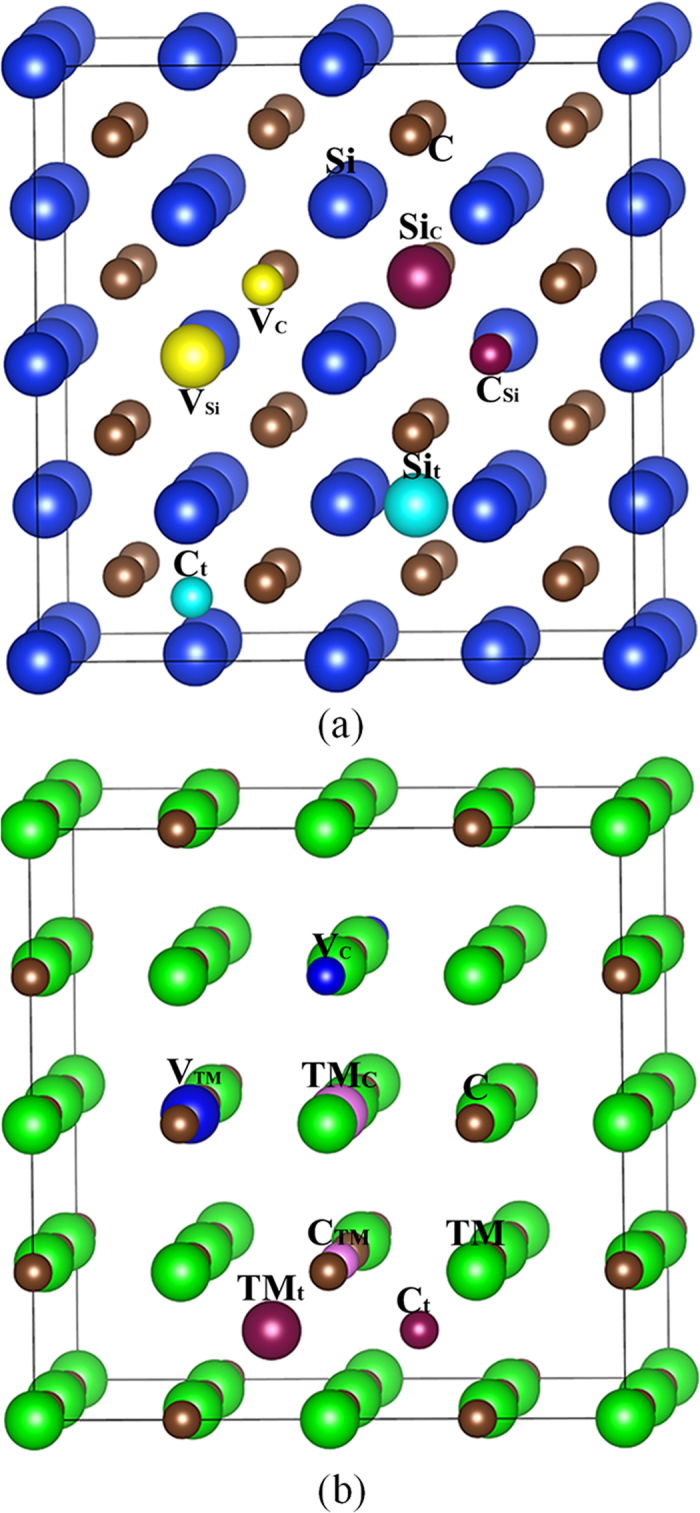
Illustration of schematic view of defects in (**a**) SiC and (**b**) TMC (TM = Zr and Ti). V_X_ (X = C, Si, Zr or Ti): X vacancy; X_t_ (X = C, Si, Zr or Ti): X interstitial occupying the tetrahedral site; X_Y_ (X, Y = C, Si, Zr or Ti) X occupying the Y lattice site.

In the literature, it has been reported that under radiation environment the created defects in MC (M = Si, Ti or Zr) carbides are mainly vacancy, interstitial and antisite defects^8, 18–20^. Thus far, it is still not clear to what degree the existence of these point defects will affect the mechanical properties of carbide compounds. Theoretically, there still lacks a systematic understanding of the thermo-physical properties of defective ZrC, TiC and SiC. In order to better understand the behaviors of carbides and improve their performance under radiation environment, it is of vital importance to perform a detailed and in-depth investigation of the mechanical and thermal properties of the MC carbides. In this study, we employed first-principles calculations based on the density functional theory to investigate the stability of different kinds of point defects, including vacancy, interstitial and antisite defects. The geometrical configurations of SiC and TMC (TM = Zr and Ti), as well as the considered point defects, are illustrated in Fig. 1(a) and (b). The computational details are described in the Method section. Based on the optimized structures, the elastic constant, elastic modulus, ductility, Debye temperature and thermal conductivity of ideal and defective carbides are all predicted. The presented results provide an atomic-level insight into the effect of point defects on the mechanical and thermal properties of these carbides, and will be useful for promoting further experimental and theoretical investigations to enhance the mechanical stability of carbide materials under irradiation.

## Results and Discussion

### Lattice parameters and formation enthalpies for ZrC, TiC and SiC

The lattice constants for bulk ZrC, TiC and SiC are calculated and summarized in Table 1, along with the available theoretical and experimental results. Our calculated lattice constants of 4.67 Å for ZrC, 4.29 Å for TiC and 4.34 Å for SiC are shown to be in excellent agreement with the theoretical and experimental results^6, 21, 22^. The formation enthalpies for carbides are obtained by $\Delta H={\mu }_{A}^{bulk}+{\mu }_{B}^{bulk}-{\mu }_{AB}^{bulk}$ ^23^. The ${\mu }_{AB}^{bulk}$ represents the chemical potential of a A-B pair in bulk AB, and ${\mu }_{A}^{bulk}$ and ${\mu }_{B}^{bulk}$ are chemical potentials of bulk A and B, respectively^23^. Our calculated formation enthalpy of ZrC is 1.77 eV/atom, which is comparable with the theoretical value of 1.89 eV/atom and experimental value of 1.92 eV/atom^24^. For the formation enthalpy of TiC, our calculated value of 1.74 eV/atom is slightly smaller than the theoretical result of 1.88 eV/atom and experimental data of 1.92 eV/atom^24^. For SiC, our calculated result of 0.56 eV/atom is much smaller than the experimental value of 1.19 eV/atom^25^, whereas it is comparable with the theoretical result of 0.67 eV/atom^26^. It is noted that the formation enthalpy of SiC is remarkably smaller than that of ZrC and TiC, indicating that the bonding of SiC is weaker than that of ZrC and TiC and they may exhibit different mechanical stability under irradiation.

**Table 1:** The calculated lattice constants (a_0_) and formation enthalpies (ΔH) for ZrC, TiC and SiC.

	ZrC		TiC		SiC	
	a_0_ (Å)	ΔH (eV/atom)	a_0_ (Å)	ΔH (eV/atom)	a_0_ (Å)	ΔH (eV/atom)
Our cal.	4.67	1.77	4.29	1.74	4.34	0.56
Other cal.	4.71^a^	1.89^a^	4.33^a^	1.88^a^	4.45^d^	0.67^e^
Exp.	4.68^b^	1.92^c^	4.32^b^	1.92^c^	4.36^d^	1.19 ^f^

^a^Ref. 6. ^b^Ref. 22. ^c^Ref. 24. ^d^Ref. 21. ^e^ Ref. 26. ^f^Ref. 25.

### The defect formation energies for ZrC, TiC and SiC

To investigate the stability of point defects, we calculated the defect formation energies based on the optimized structures. For MC (M = Si, Zr and Ti) carbides, the defect formation energy (${E}_{f}$) is calculated by ${E}_{f}={E}_{def}-{E}_{undef}+{\sum }_{i}\Delta {n}_{i}{\mu }_{i}$ ^27^. Here, ${E}_{def}$ is the total energy of defective simulation cell after relaxation, ${E}_{undef}$ is the total energy of the ideal supercell, $\Delta {n}_{i}$ is the change in the number of species *i*(*i* = Zr, Ti, Si, or C) and *μ*_*i*_ is the chemical potential of species *i*, which is defined as the total energy of its bulk state. The defect formation energies are presented and compared with other theoretical results in Table 2. For ZrC, our calculated defect formation energies are generally in good agreement with the *ab initio* calculations of Kim *et al*.^28^, with an exception of the carbon and zirconium interstitials occupying the tetrahedral site. The formation energies of 3.26 eV for C interstitial and 8.01 eV for Zr interstitial are much smaller than the respective values of 3.82 and 8.72 eV in the study of Kim et al.^28^. This is mainly due to the fact that the Zr (or C) interstitial occupies the tetrahedral site that is surrounded by four Zr (or C) in our study, whereas the tetrahedral Zr (or C) are four-coordinated with two Zr atoms and two C atoms in Kim’s study. Among the point defects, the V_C_ (carbon vacancy) defect has the lowest formation energy and the C_Zr_ antisite (carbon occupying the zirconium lattice site) defect is the most difficult to form. As compared with ZrC, the defect formation energies in TiC are generally smaller, except the C tetrahedral interstitial defect, whereas the relative stability of the defects in both carbides shows very similar character. As for SiC, our calculated defect formation energies are generally comparable with the available theoretical results^29–31^. Comparing the defect formation energies in these three carbides, we find that the V_C_ defect is the most preferable in ZrC and TiC, as indicated by the lowest formation energies, while in SiC the C_Si_ antisite defect is slightly more favorable than the V_C_, and the stability of Si_C_ antisite is comparable. These results suggest that antisite defects are relatively more easily to form in SiC than TMCs. However, both the carbon vacancy and interstitial defects are more difficult to form in SiC, since the corresponding formation energies in this compound are much higher than those in TiC and ZrC.

**Table 2:** The defect formation energies (eV) for MC (M = Zr, Ti and Si).

Defect type	Defect formation energy	
	**ZrC**	
	**Our Cal.**	**Other Cal.**
V_C_	1.08	0.93^a^
V_Zr_	8.98	8.83^a^
C_t_	3.26	3.82^a^
Zr_t_	8.01	8.72^a^
C_Zr_	13.28	13.00^a^
Zr_C_	9.56	9.56^a^
	**TiC**	
	**Our Cal**.	O**ther Cal**.
V_C_	0.80	—
V_Ti_	7.03	—
C_t_	3.59	—
Ti_t_	7.01	—
C_Ti_	12.83	—
Ti_C_	9.22	—
	**SiC**	
	**Our Cal**.	**Other Cal**.
V_C_	3.64	3.74^b^ 3.63^c^
V_Si_	8.44	8.38^b^ 7.48^c^
C_t_	6.34	5.84^d^
Si_t_	7.58	7.02^b^ 7.04^c^
C_Si_	3.56	3.28^b^ 3.48^c^
Si_C_	3.81	4.43^b^ 4.02^c^

V_X_ (X = C, Si, Zr or Ti): X vacancy; X_t_ (X = C, Si, Zr or Ti): X interstitial occupying the tetrahedral site; X_Y_ (X, Y = C, Si, Zr or Ti): X occupying the Y lattice site. ^a^Ref. 28. ^b^ref. 30. ^c^Ref. 29. ^d^Ref. 31.

In order to consider the effect of chemical environment on the defect formation energies (*E*_*f*_), we further calculate them by ref. 23

1 $${E}_{f}={E}_{def}-\frac{1}{2}({n}_{M}+{n}_{C}){\mu }_{MC}^{bulk}-\frac{1}{2}({n}_{M}-{n}_{C})({\mu }_{M}^{bulk}-{\mu }_{C}^{bulk})-\frac{1}{2}({n}_{M}-{n}_{C})\Delta \mu $$

and

2 $$\Delta \mu =({\mu }_{M}-{\mu }_{C})-({\mu }_{M}^{bulk}-{\mu }_{C}^{bulk}).$$

**Figure 2:**
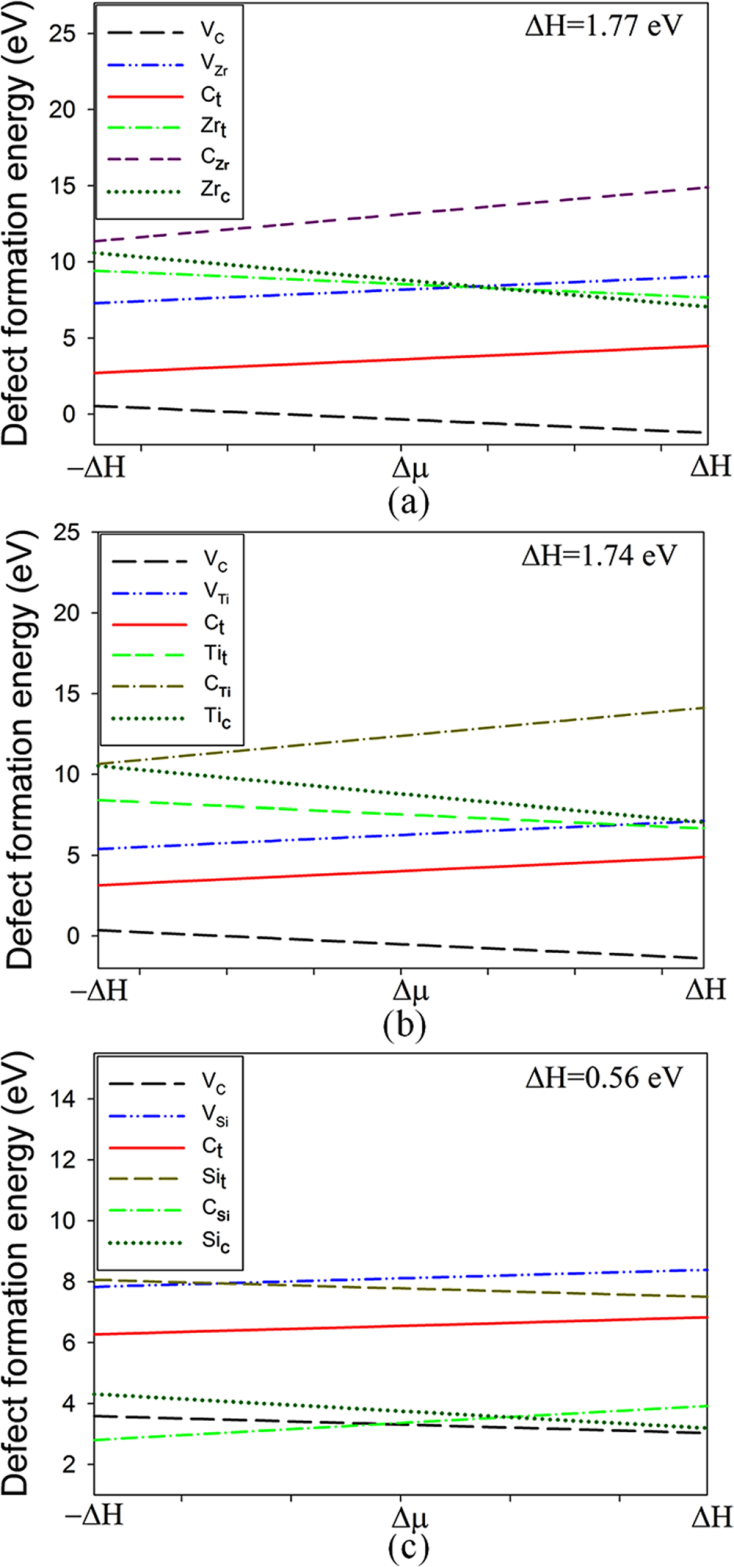
The defect formation energies as a function of chemical potential difference (Δμ) in (a) ZrC; (b) TiC; (c) SiC. The ΔH and –ΔH correspond to M-rich and C-rich conditions, respectively. V_X_ (X = C, Si, Zr or Ti): X vacancy; X_t_ (X = C, Si, Zr or Ti): X interstitial occupying the tetrahedral site; X_Y_ (X, Y = C, Si, Zr or Ti) X occupying the Y lattice site.

Here, *E*_*def*_ is the total energy of defective simulation cell after relaxation. The number of M and C atoms are denoted by *n*_*M*_ and *n*_*C*_, and Δ*μ* is the chemical potential difference, which is limited by the formation enthalpy of the carbides and varies from −Δ*H* (C-rich) to Δ*H* (M-rich). The defect formation energies as a function of chemical potential difference are plotted in Fig. 2. It turns out that the preference of V_C_ defect in ZrC and TiC is independent of the chemical environment. During the whole range of Δ*μ*, the V_C_ defect is always the most favorable and its formation energy is significantly smaller than other defects. The stability of C_t_ also does not depend on the chemical potential of carbon. It is less stable than the V_C_ defect, while its formation energy is smaller than the TM vacancy, interstitial and antisite defects. Among all the defects, the C_TM_ antisite defect shows the highest formation energy and is the most difficult to form. Due to the difference in the crystal structure and chemical bonding, SiC exhibits very different character in the defect stability, for which the relative stability of defects is chemical environment dependent. Under C-rich condition, the C_Si_ antisite defect is dominant; however, under Si-rich condition, the V_C_ and Si_C_ defects are more favorable. This is consistent with the *ab initio* molecular dynamics simulation of low energy radiation responses of carbides, in which the created defects in SiC are mainly carbon vacancy and antisite defects, whereas damage end states in TiC and ZrC generally consist of carbon Frenkel pairs and very few antisite defects are created^8^.

### The elastic constants and elastic moduli for ideal and defective carbides

The elastic constants (C_ij_) determine the response of the crystal to external forces, and provide important information about the stability, stiffness and hardness of materials. For cubic systems, there are three independent elastic constants, i.e., C_11_, C_12_ and C_44_, where C_11_ represents the uniaxial deformation along the [001] direction, C_12_ is the pure shear stress at (110) crystal plane along the [110] direction and C_44_ is a pure shear deformation on the (100) crystal plane^32^. The calculated elastic constants for ideal carbides are summarized in Table 3, along with the theoretical^6, 33^ and experimental^34–37^ values for comparison. The calculated results of C_11_ = 460.2 GPa, C_12_ = 118.1 GPa, C_44_ = 138.9 GPa for ZrC, C_11_ = 497.5 GPa, C_12_ = 143.7 GPa, C_44_ = 143.9 GPa for TiC, are generally comparable with the experimental results^35, 36^ and other theoretical^6^ results. As for SiC, C_11_, C_12_ and C_44_ are calculated to be 383.3, 125.2 and 239.6 GPa, respectively, which agree well with the theoretical^33^ and experimental^37^ values. It is noted that the C_11_ of SiC is 76.9 and 114.2 GPa smaller than that of ZrC and TiC, respectively, whereas the C_44_ of SiC is 100.7 GPa larger than ZrC and 95.7 GPa larger than TiC. Obviously, the resistance to uniaxial deformation along the [001] direction and shear deformation on the (100) plane for SiC is distinct from that for ZrC and TiC, and this may cause different mechanical and between SiC and TMCs.

**Table 3:** The elastic constants (GPa), bulk modulus B (GPa), Young’s modulus E (GPa) and shear modulus G (GPa) for pristine ZrC, TiC and SiC.

	C_11_	C_12_	C_44_	B	E	G
	ZrC					
Our Cal.	460.2	118.1	138.9	232.2	372.3	150.9
Other Cal.	445.6^a^	103.5^a^	137.8^a^	217.5^a^ 235^b^	406.6^a^ 400^b^	150.3^a^ 164^b^
Exp.	470^c^	100^c^	160^c^	208^d^	386^d^	162^d^
	TiC					
Our Cal.	497.5	143.7	143.9	261.6	391	156.3
Other Cal.	532^a^	115.6^a^	206.9^a^	251^a^ 257^e^	481^a^ 401^e^	205^a^ 162^e^
Exp.	500^f^	113^f^	175^f^	242^f^	437^f^	182^f^
	SiC					
Our Cal.	383.3	125.2	239.6	211.3	432.9	186.9
Other Cal.	384.5^g^	121.5^g^	243.3^g^	209^g^	437.6^g^	190.1^g^
Exp.	390^h^	142^h^	256^h^	225^h^	448^h^	192^h^

^a^Ref. 6. ^b^Ref. 38. ^c^Ref. 35. ^d^Ref. 34. ^e^Ref. 41. ^f^Ref. 36. ^g^Ref. 33. ^h^Ref. 37.

**Table 4:** The elastic constants (GPa) for ideal and defective MC (M = Zr, Ti and Si).

		Ideal	V_C_	V_M_	C_t_	M_t_	C_M_	M_C_
ZrC	C_11_	460.2	459.9	442.2	462.8	391.6	449.3	446.4
	C_12_	118.1	107.5	111.9	114.7	123.6	112.9	98.2
	C_44_	138.9	143.8	131.8	140.4	141.6	136.2	86.3
TiC	C_11_	497.5	503.3	487.3	505.0	433.5	481.5	494.9
	C_12_	143.7	129.4	137.3	139.4	149.9	141.2	122.3
	C_44_	143.9	155.1	139.1	155.2	157.8	154.9	88.2
SiC	C_11_	383.3	337.4	328.4	364.2	338.1	390.1	369.3
	C_12_	125.2	132.0	121.4	127.1	114.0	125.6	120.9
	C_44_	239.6	81.9	186.5	215.3	168.0	240.9	225.6

V_X_ (X = C, Si, Zr or Ti): X vacancy; X_t_ (X = C, Si, Zr or Ti): X interstitial occupying the tetrahedral site; X_Y_ (X, Y = C, Si, Zr or Ti): X occupying the Y lattice site.

Based on the optimized structures, the elastic constants for defective ZrC, TiC and SiC are calculated and summarized in Table 4. As compared with the pristine states, the C_44_ values of ZrC and TiC containing TM_C_ antisite defects are reduced considerably, i.e., ~38%. The existence of TM_C_ antisite defects also decreases the C_12_ values by 16.8% and 14.9% for ZrC and TiC, respectively. On the other hand, the TM interstitial defects decrease the C_11_ of ZrC and TiC by 14.9% and 12.9%, respectively, and increase the C_44_ of TiC by 9.6%. As the most favorable defect in ZrC and TiC, the V_C_ defect decreases the C_12_ of ZrC and TiC by 9% and 10%, respectively. Generally, the TM_C_ antisite and TM_t_ interstitial defects, which are not preferable for TMC under irradiation, have more significant effects on the elastic constants, and the dominant V_C_ defect has smaller influences. The situation in SiC is found to be very different. In this carbide, the reduction in the C_44_ value by the dominant V_C_ defect is as high as 65.8%. The decrease of 12% in C_11_ caused by the V_C_ defect is also not negligible. Besides, the V_Si_ and Si_t_ also decrease the C_44_ significantly, i.e., 22% and 30%, respectively. As for the C_Si_ and Si_C_ antisite defects, the influences are relatively much smaller. Obviously, the defects of V_C_, V_Si_ and Si_t_ affect the elastic constants of SiC remarkably. These results suggest that the existence of the TM_C_ antisite and TM_t_ interstitial defects in TMC carbides, and the V_C_, V_Si_ and Si_t_ defects in SiC generally weakens the resistance to uniaxial and shear deformation.

The bulk, shear and Young’s moduli can be estimated using Voigt-Reuss-Hill (VRH) approximation, which is an average of the lower bound of Voigt and upper bound of Reuss, and can provide a good estimation of the mechanical properties from the elastic constants. The elastic moduli and Poisson’s ratio are defined as follows:

3 $$B=\frac{1}{3}({C}_{11}+2{C}_{12}),$$

4 $${G}_{V}=\frac{1}{5}({C}_{11}-{C}_{12}+3{C}_{44}),$$

5 $${G}_{R}=\frac{5({C}_{11}-{C}_{12}){C}_{44}}{4{C}_{44}+3({C}_{11}-{C}_{12})},$$

6 $${G}_{VRH}=\frac{1}{2}({G}_{V}+{G}_{R}),$$

7 $$E=\frac{9{B}_{VRH}{G}_{VRH}}{(3{B}_{VRH}+{G}_{VRH})},$$

and

8 $$\sigma =\frac{(3{B}_{VRH}-2{G}_{VRH})}{[2(3{B}_{VRH}+{G}_{VRH})]}.$$

Here, *G*_*V*_, *G*_*R*_ and *G*_*VRH*_ are the shear modulus calculated by Voigt, Reuss and Voigt-Reuss-Hill approximation, respectively, B is the bulk modulus, E is the Young’s modulus, and *σ* is the Poisson’s ratio. Our calculated elastic moduli for ideal carbides together with the available theoretical and experimental results are summarized in Table 3. For ZrC, our calculated results are B = 232.2 GPa, E = 372.3 GPa and G = 150.9 GPa. In the literature, Yang *et al*. employed the similar method and determined the elastic moduli of ZrC to be 235, 400 and 164 GPa for B, E and G, respectively^38^, which are comparable with our results. Liu *et al*. carried out DFT calculations with CASTEP code^39^ and ultrasoft pseudopotentials^40^, and also obtained similar results, i.e., B = 217.5 GPa, E = 406.6 GPa and G = 150.3 GPa^6^. As for TiC, our calculated bulk, Young’s and shear moduli are 261.6, 391 and 156.3 GPa, respectively, which are in good agreement with the study of Lu *et al*.^41^, where the same code and similar computational parameters have been employed. Comparing our results with the calculations of Liu *et al*.^6^, we find that their values of B = 251 GPa, G = 481 GPa and E = 205 GPa differ a lot from our results. The large discrepancy in the Young’s and shear moduli between Liu’s study and this work as well as the work of Lu *et al*. is probably due to the difference in the force convergence criteria. In this work and Lu’s study, the convergence criteria for force is set to be 1 × 10^−5^ eV/Å and 1 × 10^−6^ eV/Å, respectively, while in Liu’s study the criteria is only 1 × 10^−2^ eV/Å. For SiC, the bulk, Young’s and shear moduli are calculated to be 211.3, 432.9 and 186.9 GPa, respectively, which are in reasonable agreement with the experimental^37^ and theoretical^33^ results.

The elastic moduli for defective carbides are summarized in Table 5, along with the relative change of respective elastic modulus. As defects are introduced into ZrC, we find that the Zr_C_ antisite defect has a remarkably negative effect on the Young’s and shear moduli, which decrease from 372.2 to 292.1 GPa and from 150.9 to 114.8 GPa, respectively. The bulk modulus is also decreased by 7.7% by the presence of Zr_C_ antisite defect. Another defect that influences the elastic moduli relatively a lot is the Zr_t_ defect, which reduces the bulk, Young’s and shear modulus by about 8.3%. As for the V_C_, V_Zr_, C_t_ and C_Zr_ defects, the influences are much smaller and even negligible in some cases. In TiC, the most significant change is found to be the Ti_C_ antisite defect, for which the decreases are as large as 21% for Young’s modulus and 23.5% for shear modulus. The Ti_t_ and Ti_C_ defects also decrease the bulk modulus by 6.6% and 5.8%, respectively. It is noticeable that the carbon vacancy and interstitial defects, which are more favorable than other defects under irradiation, have positive effects on the shear modulus, although the effect is not significant. In SiC, the influences of point defect on the elastic moduli are also generally negative. The most pronounced change is found for SiC with V_C_ defect, for which the Young’s modulus of 234 GPa and shear modulus of 89.6 GPa are remarkably smaller than the respective values of 433 GPa and 187 GPa for the ideal state. The silicon vacancy and interstitial defects also affect the elastic moduli largely, which cause the bulk, Young’s and shear modulus to decrease by 9.9-10.7%, 18.9-21% and 21.2-23.6%, respectively. On the other hand, the Si_C_ antisite defect also leads to a reduction of 12.4% in the bulk modulus.

**Table 5:** The bulk modulus B (GPa), Young’s modulus E (GPa), shear modulus G (GPa) for ideal and defective MC (M = Zr, Ti and Si), along with the relative change of the bulk modulus (ΔB), Young modulus (ΔE) and shear modulus (ΔG).

	Ideal	V_C_	V_M_	C_t_	M_t_	C_M_	M_C_
ZrC							
B	232.2	224.9	222.0	230.7	212.9	225.1	214.3
ΔB (%)		−3.1	−4.3	−0.6	−8.3	−3.1	−7.7
E	372.2	380.1	355.7	375.9	341.5	364.6	292.2
ΔE (%)		2.2	−4.4	1.0	−8.3	−2.0	−21.5
G	150.9	155.9	144.2	152.9	138.50	148.2	114.8
ΔG (%)		3.3	−4.5	1.3	−8.2	−1.8	−24
TiC							
B	261.6	254.1	251.9	261.3	244.4	254.6	246.5
ΔB (%)		−2.9	−3.7	−0.1	−6.6	−2.7	−5.8
E	391.0	411.2	381.8	410.4	376.0	398.6	308.8
ΔE (%)		5.0	−2.4	4.9	−3.8	1.9	−21
G	156.3	167.1	153.0	165.7	151.2	160.8	119.6
ΔG (%)		7	−2.1	6.0	−3.3	2.9	−23.5
SiC							
B	211.3	200.5	190.4	206.1	188.7	213.7	185.0
ΔB (%)		−5.1	−9.9	−2.4	−10.7	1.2	−12.4
E	432.9	233.9	351.2	398.9	342.1	438.6	412.7
ΔE (%)		−46	−18.9	−7.8	−21	1.3	−4.7
G	186.9	89.6	147.3	169.5	142.8	189.4	177.6
ΔG (%)		−52.1	−21.2	−9.3	−23.6	1.3	−5.0

V_X_ (X = C, Si, Zr or Ti): X vacancy; X_t_ (X = C, Si, Zr or Ti): X interstitial occupying the tetrahedral site; X_Y_ (X, Y = C, Si, Zr or Ti): X occupying the Y lattice site.

**Figure 3:**
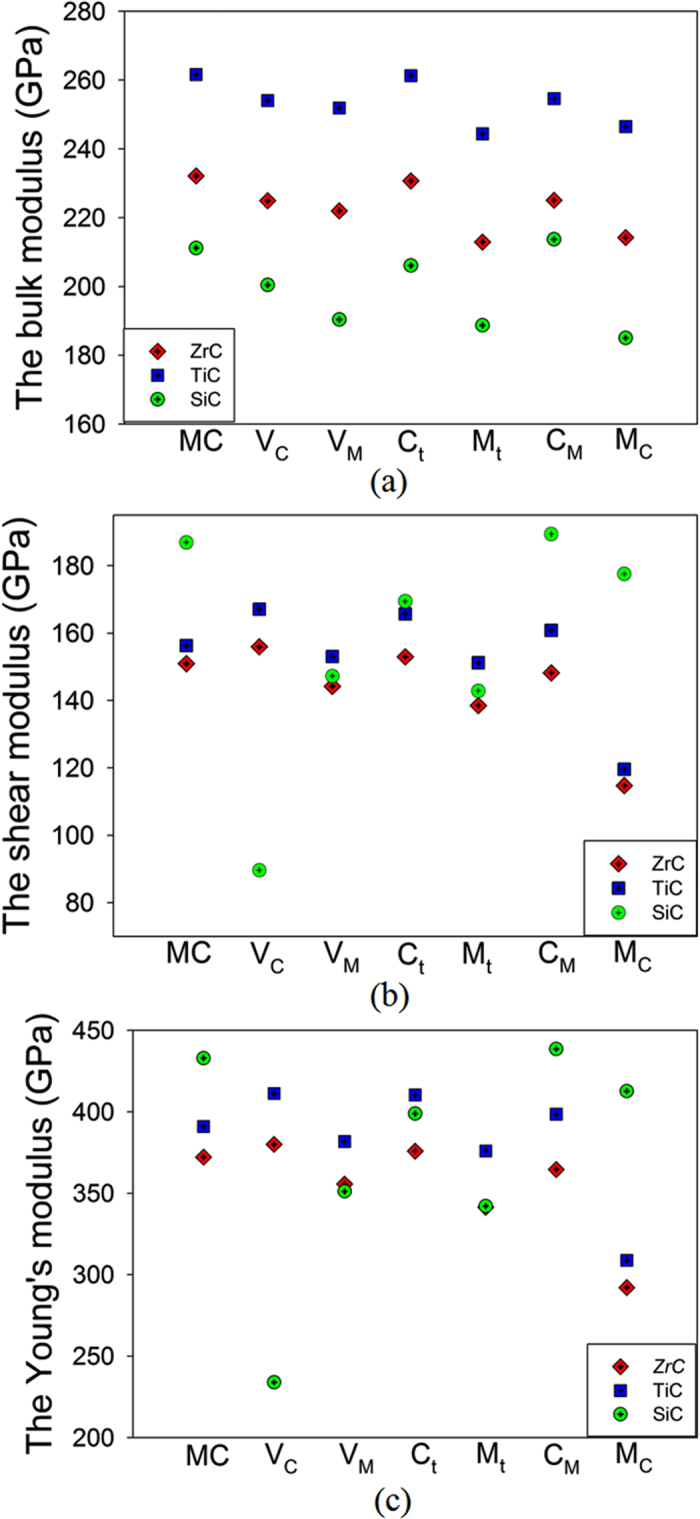
The calculated (**a**) bulk modulus (B), (**b**) shear modulus G and (**c**) Young’s modulus for ideal and defective MC (M = Zr, Ti and Si). V_X_ (X = C, Si, Zr or Ti): X vacancy; X_t_ (X = C, Si, Zr or Ti): X interstitial occupying the tetrahedral site; X_Y_ (X, Y = C, Si, Zr or Ti) X occupying the Y lattice site.

The variation of the elastic moduli for the ideal and defective carbides is illustrated in Fig. 3. It is noted that the bulk moduli for defective SiC are generally smaller than those for defective ZrC and TiC. As for the shear and Young’s moduli, the most significant changes are found for SiC with V_C_ defect and TMCs with TM_C_ antisite defect. Considering that the V_C_ defect dominates in TMCs, and the V_C_, C_Si_ and Si_C_ defects are the predominant defects in SiC, we propose that under radiation environment, the elastic moduli of SiC may be more damaged than ZrC and TiC, and the performance of SiC may be deteriorated to a greater extent. As one of the most important coating in Tristructural-isotropic particles and gas-cooled fission reactors, SiC provides most of the structural strength and dimensional stability. The ZrC and TiC, with larger bulk modulus and smaller changes in the Young’s and shear moduli under radiation environment, are thus promising alternatives to SiC.

### Ductility and Poisson’s ratio of ideal and defective ZrC, TiC and SiC

Pugh proposed the ratio of B/G to empirically predict the ductility of material^42^. The critical value which separates ductile and brittle materials is around 1.75, i.e., if B/G ratio is smaller than 1.75, the material demonstrates brittleness; otherwise, it behaves in a ductile manner^42^. Besides B/G ratio, the Cauchy pressure (C.P.), which is calculated by the equation C.P. = C_12_-C_44_, can also be used to indicate the ductility of material. If the C.P. is positive, the material is characterized to be ductile; otherwise, it is brittle^43^.

The calculated B/G ratio and Cauchy pressure for the ideal and defective carbides are presented in Table 6. The B/G ratio of 1.54 for ZrC is comparable with the theoretical value of 1.45 reported by Liu *et al*.^6^, and both results are larger than the experimental value of 1.31^34^. As compared with the value of 1.54 for ideal ZrC, the B/G ratios for ZrC with V_Zr_, C_t_, Zr_t_ and C_Zr_ defects are generally comparable, whereas the values of 1.44 for V_C_ and 1.87 for Zr_C_ are smaller and larger, respectively. The Cauchy pressure of -36.3 GPa for ZrC with V_C_ defect and 11.9 GPa for ZrC with Zr_C_ antisite defect are also smaller and larger than the value of −20.8 GPa for ideal ZrC, respectively. These results indicate that the ZrC with Zr_C_ antisite defect is more ductile than the ideal state, and the ZrC with V_C_ defect is relatively more brittle. As for TiC, the B/G ratio is 1.67, which is larger than the theoretical value of 1.22^6^ and the experimental data of 1.33^36^. Our calculated bulk modulus of 261.6 GPa is comparable with Liu’s result of 251 GPa, whereas large discrepancy exist between Liu’s overestimated shear modulus of 205 GPa and our shear modulus of 156.3 GPa, which leads to the different B/G ratios. It is noted that the B/G ratios for defective TiC with V_C_, V_Ti_, C_t_, Ti_t_ and C_Zr_ defects are smaller than the value of 1.68 for ideal TiC, while the value of 2.06 for the case of Ti_C_ defect is considerably larger. Also, the Cauchy pressures for the defective TiC are smaller than the ideal state except Ti_C_ antisite defect. These results suggest that the presence of Ti_C_ antisite defect results in better ductility. In the case of SiC, the B/G ratio of 1.13 agrees well with the theoretical and experimental values of 1.17^37, 44^. It is noted that B/G ratios for defective SiC are generally larger than the value of 1.13, with an exception of C_Si_ defect. Especially, the SiC with V_C_ defect exhibits a much larger value of 2.24. Obviously, the introduction of point defects in SiC generally enhances its ductility, which can also be confirmed by the calculated results of Cauchy pressure. The defective ZrC and TiC generally exhibit larger B/G ratio and Cauchy pressure than defective SiC (except the V_C_ state). Particularly, the Si_C_ and C_Si_ antisite defective states, which dominate in SiC under irradiation, are more brittle than defective ZrC and TiC. These results demonstrate that when these three carbides are exposed to radiation environment, the ZrC and TiC generally have better ductility than SiC.

**Table 6:** The B/G ratio, Cauchy pressure C.P (GPa) and Poisson’s ratio (σ) for ideal and defective MC (M = Zr, Ti and Si).

	Ideal	V_C_	V_M_	C_t_	M_t_	C_M_	M_C_
ZrC							
B/G	1.54	1.44	1.54	1.51	1.54	1.52	1.87
	1.45^a^ 1.31^b^						
C.P	−20.8	−36.3	−19.9	−25.7	−17.9	−23.2	11.9
σ	0.23	0.22	0.23	0.23	0.23	0.23	0.27
	0.19^a^ 0.19^b^						
TiC							
B/G	1.67	1.52	1.65	1.58	1.62	1.58	2.06
	1.22^a^ 1.33^c^						
C.P	−0.19	−25.7	−4.8	−15.8	−7.9	−13.7	34.1
σ	0.25	0.23	0.25	0.24	0.24	0.24	0.29
	0.18^a^ 0.20^c^						
SiC							
B/G	1.13	2.24	1.29	1.22	1.32	1.13	1.15
	1.17^d^ 1.17^e^						
C.P	−114.4	50.2	−65.1	−88.2	−53.9	−115.4	−104.7
σ	0.16	0.31	0.19	0.18	0.20	0.16	0.16
	0.17^d^ 0.16^e^						

V_X_ (X = C, Si, Zr or Ti): X vacancy; X_t_ (X = C, Si, Zr or Ti): X interstitial occupying the tetrahedral site; X_Y_ (X, Y = C, Si, Zr or Ti): X occupying the Y lattice site. ^a^Ref. 6. ^b^Ref. 34. ^c^Ref. 36. ^d^Ref. 44. ^e^Ref. 3.

As is well known, the Poisson’s ratio (σ) can be regarded as an indicator to measure the degree of directionality of bonds. The value of σ is about 0.1 for strongly covalent crystals, whereas it is greater than or equal to 0.25 for ionic materials^45^. As shown in Table 6, the ratios are determined to be 0.23 for ZrC, 0.25 for TiC and 0.16 for SiC, which are consistent with the experimental^34, 36, 37^ and other theoretical^6, 44^ results. It is noted that the Poisson’s ratio for SiC is smaller than that of ZrC and TiC, meaning that the <Si-C> bond is more covalent than the <Zr-C> and <Ti-C>. This is consistent with our previous calculations, which demonstrated that the nature of <Si-C> is covalent, while the <Ti-C> and <Zr-C> bands are a mixture of covalent, ionic and metallic bands. For ZrC and TiC, it is shown that the σ values for defective states are generally comparable with the ideal states. One exception is the TMC with TM_C_ antisite defects, for which the σ values are much larger and the <TM-C> bonds are more ionic. As for SiC, the σ values for Si_C_ and C_Si_ antisite defects are comparable with the ideal state, and other defective states have larger σ values, particularly the SiC with V_C_ defect, indicating that the vacancy and interstitial defects reduce the covalency of the <Si-C> bond. Comparing the σ values for defective ZrC, TiC and SiC, we find that the defective ZrC and TiC generally have larger Poisson’s ratios, except for SiC with the V_C_. These results suggest that the <TM-C> bonds are less covalent than the <Si-C> bond. Since the defective ZrC and TiC are more ductile than SiC, we propose that the better ductility of ZrC and TiC may be resulted from the less covalency of the <TM-C> bonds.

### The thermodynamic properties of pristine and defective carbides

The Debye temperature is often used to characterize many properties of materials, such as thermal vibration of atoms, heat capacity, and thermal expansion coefficient^46^. It can be calculated by ref. 47

9 $${\Theta }_{D}=\frac{h}{k}{[\frac{3n}{4\pi }(\frac{{N}_{A}\rho }{M})]}^{\frac{1}{3}}{\nu }_{m},$$

10 $${\nu }_{m}={[\frac{1}{3}(\frac{2}{{\nu }_{s}^{3}}+\frac{1}{{\nu }_{l}^{3}})]}^{-\frac{1}{3}},$$

11 $${\nu }_{l}=\sqrt{\frac{(B+4/3G)}{\rho }}$$

and

12 $${\nu }_{s}=\sqrt{\frac{G}{\rho }}.$$

Here, Θ_*D*_ is the Debye temperature, *h* and *k* are the Planck’s and Boltzmann’s constant, respectively, *n* is the number of atoms per unit cell, *N*_*A*_ is the Avogadro’s number, *M* is the unit-cell molecular weight and $\rho$ is the density. In the equations (10)–(12), *v*_*m*_, *v*_*l*_ and *v*_*s*_ are the average sound wave velocity, longitudinal sound wave velocity and shear sound wave velocity, respectively. The calculated average sound wave velocity and Debye temperature for pristine and defective carbides are summarized in Table 7, together with the available experimental^48–50^ and theoretical^6, 44, 51^ results. It is noted that the Zr_C_ and Ti_C_ antisite defects have the most pronounced negative effects on the average sound wave velocity, as indicated by the decrease from 5316.6 to 4657.7 m/s for ZrC and from 6277.7 to 5508.1 m/s for TiC. As for SiC, the most significant change is found for the V_C_ defect, for which there is a sharp decrease in the average sound wave velocity from 1146.3 to 807.2 m/s.

**Table 7:** The average sound wave velocity ν_m_ (m/s), Debye temperature Θ_D_ (K), phonon thermal conductivity *κ*_*p*_ (W/mk) and electronic thermal conductivity *κ*_*e*_(W/mk) for ideal and defective MC (M = Zr, Ti and Si).

	Ideal	V_C_	V_M_	C_t_	M_t_	C_M_	M_C_
ZrC							
${\upsilon }_{m}$	5316.6	5395.4	5195.8	5349.6	5092.6	5266.3	4657.7
Θ_D_	672.6	682.5	657.3	676.7	644.2	666.2	589.2
	669.6^a^713.8^b^ 699*^c^						
${\kappa }_{p}^{Clark}$	1.63	1.65	1.59	1.64	1.56	1.61	1.44
${\kappa }_{p}^{Cahill}$	1.78	1.79	1.74	1.78	1.70	1.76	1.59
${\kappa }_{e}$	5.03	4.78	4.56	4.93	5.22	4.72	5.02
TiC							
${\upsilon }_{m}$	6277.7	6476.1	6209.2	6454.4	6169.1	6359.3	5518.0
Θ_D_	863.4	890.7	853.9	887.7	848.4	874.6	758.9
	980.7^a^946.3^b^ 940*^d^						
${\kappa }_{p}^{Clark}$	2.29	2.34	2.26	2.34	2.24	2.31	2.03
${\kappa }_{p}^{Cahill}$	2.50	2.56	2.47	2.56	2.45	2.52	2.25
${\kappa }_{e}$	6.02	5.11	5.13	5.21	5.26	5.76	5.37
SiC							
${\upsilon }_{m}$	8387.5	5906.1	7471.0	8001.7	7362.1	8443.1	8157.6
Θ_D_	1146.3	807.2	1020.1	1093.6	1006.2	1153.9	1114.9
	1111^e^1123*^g^						
${\kappa }_{p}^{Clark}$	2.93	2.16	2.64	2.81	2.61	2.95	2.86
${\kappa }_{p}^{Cahill}$	3.19	2.40	2.87	3.06	2.83	3.21	3.08
${\kappa }_{e}$	—	0.85	0.15	—	—	—	—

V_X_ (X = C, Si, Zr or Ti): X vacancy; X_t_ (X = C, Si, Zr or Ti): X interstitial occupying the tetrahedral site; X_Y_ (X, Y = C, Si, Zr or Ti): X occupying the Y lattice site. Note: *experimental result. ^a^Ref. 6. ^b^Ref. 51. ^c^Ref. 48. ^d^Ref. 49. ^e^Ref. 44. ^f^Ref. 50.

The Debye temperatures are determined to be 672.6 K for ZrC and 863.3 K for TiC, which are comparable with the theoretical^6, 51^ and experimental^48, 49^ results. It is noted that the Zr_C_ and Ti_C_ antisite defects cause a reduction of 83.3 and 104.5 K in the Debye temperature of ZrC and TiC, respectively. As for other defects, the influences are minor and even negligible in some cases. For SiC, the calculated Debye temperature is 1146.3 K, which compares well with the theoretical value of 1111 K^44^ and experimental value of 1123 K^50^. The most pronounced change is found for SiC with V_C_ defect, for which the Debye temperature of 807.2 K is remarkably smaller than the 1146.3 K for the ideal state. The V_Si_ and Si_t_ defects also affect the Debye temperature largely, which cause it to decrease to 1020.1 and 1006.2 K, respectively. Generally, the material with lower Debye temperature has weaker chemical bonding and larger thermal expansion coefficient. Comparing the Debye temperature for pristine and defective carbides, we find that the Debye temperatures of the ideal and defective SiC are generally larger than those of transition metal carbides (except the V_C_ defective state), suggesting that the SiC will have smaller thermal expansion coefficient. The carbide materials, which are used as fuel-cladding and structural materials in nuclear reactors, need to satisfy the criterion of small thermal expansion to prevent coating failure. The ZrC and TiC, with better physical and thermal properties such as larger bulk moduli and better ductility, are promising alternatives to SiC. However, the lower Debye temperature for ZrC and TiC than SiC is not beneficial to their applications. It is thus necessary to improve the Debye temperature of ZrC and TiC for these applications as fuel-cladding and structural materials in nuclear reactors.

**Figure 4:**
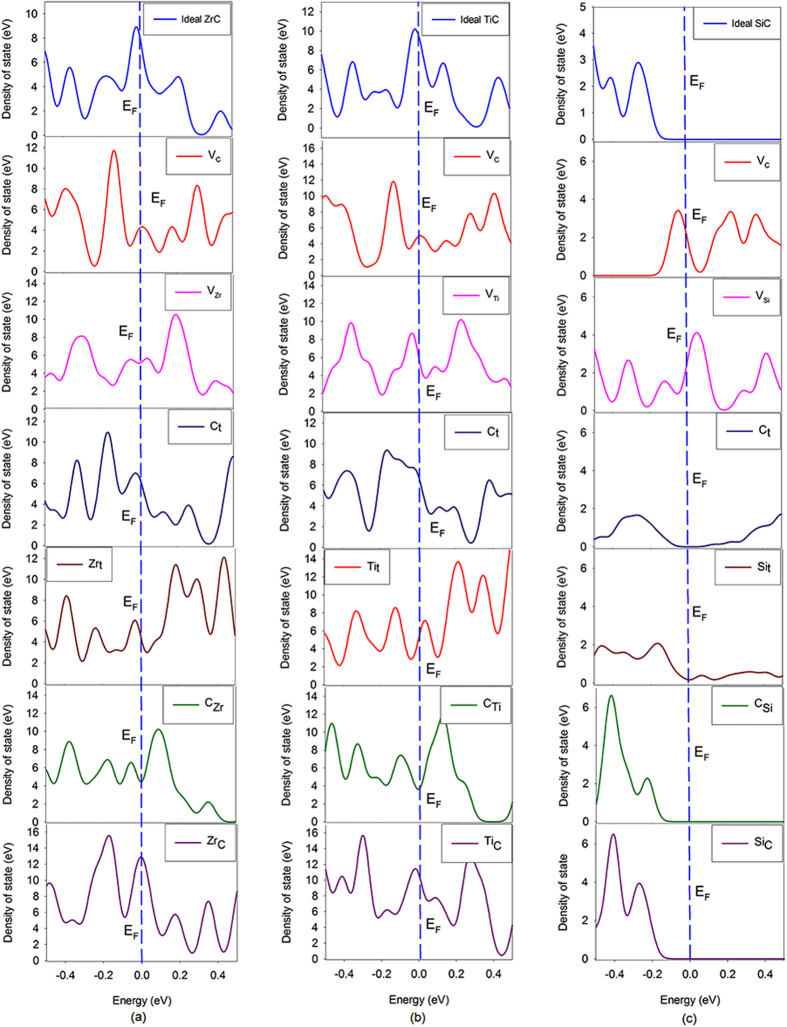
The total density of state distribution for ideal and defective carbide compounds. (**a**) ZrC, (**b**) TiC and (**c**) SiC. E_F_ represents the Fermi level and is set to be zero.

We further carry out density of state (DOS) analysis of the ideal and defective carbides. The total DOS distribution around the Fermi level for all carbides is illustrated in Fig. 4. As seen in Fig. 4, the SiC is a semiconductor while the ZrC and TiC compounds are of metallic character. It is shown that the introduction of point defects into ZrC and TiC does not change their metallic nature, and there are electrons distributing on the Fermi level of all defective states, while for SiC the defective states are still of the semiconducting character, except theV_C_ and V_Si_ defective states. Generally, the thermal conductivity of semiconductors and metals are mainly contributed by phonons and electrons, respectively. The minimum high-temperature phonon part of thermal conductivity can be obtained by Clarke’s model^52^, i.e. ${\kappa }_{p}^{Clarke}=0.87\,k{M}^{\frac{-2}{3}}{E}^{\frac{1}{2}}{\rho }^{\frac{1}{6}}$, and Cahill’s model^53^, i.e., ${\kappa }_{p}^{Cahill}=\frac{k}{2.48}{(\frac{\rho {N}_{A}}{M})}^{\frac{2}{3}}({\nu }_{l}+2{\nu }_{s}).$ Here, *k* is the Boltzmann’s constant, *M* is the unit-cell molecular weight, *E* is the Young’s modulus, *ρ* is the density, *N*_*A*_ is the Avogadro’s number, *v*_*l*_ and *v*_*s*_ are the longitudinal and shear sound wave velocity, respectively. The electronic thermal conductivity for metallic state of carbides is calculated by ${\kappa }_{e}=L\sigma T$ ^54^, where *L* is the Lorenz number and *σ* is the electrical conductivity. Here, the Lorenz number is taken to be 2.45 × 10^−8^(*W*Ω/*K* ^2^) and the electrical conductivity is obtained from the imaginary part of the dielectric function^55^. The calculated phonon thermal conductivity for all carbides and electronic thermal conductivity for metallic states are all summarized in Table 7. As expected, the thermal conductivity of ZrC and TiC are mainly contributed by electrons and the contribution of phonons is nearly half of the contribution of electrons. As for SiC with V_C_ and V_Si_, although electrons are distributing on the Fermi level, the contribution of electrons to the thermal conductivity is slight and the thermal conductivity is dominated by the phonons. Comparing the total thermal conductivity of all carbides, the thermal conductivity of ZrC and TiC are much larger than that of SiC, implying that under radiation environment the ZrC and TiC will exhibit superior heat conduction ability than the SiC.

## Conclusions

In this work, a density functional theory study is performed to compare the mechanical and thermal properties of defective ZrC, TiC and SiC. The calculated defect formation energies show that the carbon vacancy is always the most preferable in ZrC and TiC, whereas in SiC the C_Si_ antisite defect is dominant under C-rich condition and the carbon vacancy and Si_C_ antisite defects are more favorable under Si-rich condition. It is shown that the carbon vacancy has minor effects on the elastic moduli, ductility and Debye temperature of ZrC and TiC, whereas its influence on the thermo-physical properties of SiC is significant. Generally, the defective ZrC and TiC have larger bulk modulus and smaller changes in the Young’s and shear moduli than SiC under radiation environment. Although the presence of defects decreases and increases the ductility of TMCs and SiC, respectively, the defective ZrC and TiC generally have larger B/G ratio and Cauchy pressure than defective SiC, and have better ductility. We also found that the thermal conductivity of defective ZrC and TiC are much larger than that of SiC. Meanwhile, the Debye temperatures for the defective SiC are generally larger than those for transition metal carbides, indicating that the SiC has smaller thermal expansion coefficient. As the alternatives to SiC as fuel-cladding and structural materials in nuclear reactors, the ZrC and TiC generally exhibit better mechanical and thermal properties than the SiC except the Debye temperature. It is thus necessary to improve the Debye temperature of transition metal carbides for their potential applications under irradiation.

## Methods

All the calculations are carried out within the density functional theory framework using the projector augmented wave method, as implemented in Vienna *Ab Initio* Simulation Package (VASP)^56^. Projector augmented-wave pseudopotentials^57^ are used to describe the interaction between ions and electrons, and the exchange-correlation effects are treated using the generalized gradient approximation (GGA) in the Perdew-Wang parameterization^58^. The Computations are based on a 2 × 2 × 2 supercell consisting of 64 atoms with a 4 × 4 × 4 k-point sampling in reciprocal space and a cutoff energy of 500 eV. The convergence criteria for total energies and forces are 10^−5^ eV and 10^−5^ eV/Å, respectively. The point defects, which are taken into account in the simulations, include vacancy, interstitial and antisite defects and the geometrical configurations of considered point defects are also illustrated in Fig. 1.
